# Do patients with schizophreniform and bipolar disorders show an intrathecal, polyspecific, antiviral immune response? A pilot study

**DOI:** 10.1186/s12987-017-0082-1

**Published:** 2017-12-07

**Authors:** Dominique Endres, Daniela Huzly, Rick Dersch, Oliver Stich, Benjamin Berger, Florian Schuchardt, Evgeniy Perlov, Nils Venhoff, Sabine Hellwig, Bernd L. Fiebich, Daniel Erny, Tilman Hottenrott, Ludger Tebartz van Elst

**Affiliations:** 1 Section for Experimental Neuropsychiatry, Department of Psychiatry and Psychotherapy, Medical Center-University of Freiburg, Faculty of Medicine, University of Freiburg, Freiburg, Germany; 2 Institute for Virology, Medical Center-University of Freiburg, Faculty of Medicine, University of Freiburg, Freiburg, Germany; 3 Department of Neurology and Neurophysiology, Medical Center-University of Freiburg, Faculty of Medicine, University of Freiburg, Freiburg, Germany; 4 Department of Rheumatology and Clinical Immunology, Medical Center-University of Freiburg, Faculty of Medicine, University of Freiburg, Freiburg, Germany; 5 Institute of Neuropathology, Medical Center-University of Freiburg, Faculty of Medicine, University of Freiburg, Freiburg, Germany; 6 Berta-Ottenstein-Programme, Faculty of Medicine, University of Freiburg, Freiburg, Germany

**Keywords:** Schizophrenia, Bipolar disorder, Antibody index, MRZ reaction, Immunological encephalopathy

## Abstract

**Background:**

We previously described inflammatory cerebrospinal fluid (CSF) alterations in a subgroup of patients with schizophreniform disorders and the synthesis of polyspecific intrathecal antibodies against different neurotropic infectious pathogens in some patients with bipolar disorders. Consequently, we have measured the prevalence of a positive MRZ reaction (MRZR)—a marker for a polyspecific, antiviral, intrathecal, humoral immune response composed of three antibody indices for the neurotropic viruses of measles (M), rubella (R), and varicella zoster (Z)—in these patients.

**Methods:**

We analyzed paired CSF and serum samples of 39 schizophreniform and 39 bipolar patients. For comparison, we used a group of 48 patients with other inflammatory neurological disorders (OIND) and a cohort of 203 multiple sclerosis (MS) patients.

**Results:**

We found a positive MRZR in two patients with schizophreniform disorders (5.1%); both suffered from schizodepressive disorders without any other signs suggestive of MS. None of the bipolar patients (0%) and four members of the OIND group (8.3%) showed a positive MRZR. In the MS cohort, a positive MRZR was found significantly more frequently [in 99 patients (48.8%)] than in the other patient groups (p > 0.001). In summary, we did not find a positive MRZR in a relevant subgroup of patients with schizophreniform or bipolar disorders.

**Conclusions:**

Our results indicate that the MRZR is highly specific to MS. Nevertheless, two schizodepressive patients also had a positive MRZR. This finding corresponds to the few MRZR-positive patients with OIND or other autoimmune disorders with central nervous involvement, implicating that the MRZR specificity for MS is high, but not 100%.

## Background

Schizophreniform and bipolar disorders are common psychiatric axis I disorders, with prevalence rates of at least 1% [1, 2]. Schizophreniform disorders are characterized by dysexecutive, amotivational, disorganized, affective, delusional, hallucinatory, or catatonic symptoms [3]. Bipolar disorders present with depressive, (hypo)manic, or mixed episodes. From a pathophysiological perspective, the primary idiopathic forms of these disorders and secondary forms with a recognizable cause can be distinguished. The primary idiopathic forms often display a familial liability associated with polygenetic vulnerability. Secondary forms can be due to different brain disorders caused by immunological (limbic encephalitis, anti-NMDA-R-encephalitis, Hashimoto’s encephalopathy, etc.), infectiological (neuroborreliosis, neurosyphilis, etc.), epileptic (paraepileptic psychosis, etc.), metabolic (Niemann Pick type c, etc.), vascular (vasculitis, etc.), traumatic (traumatic brain injury), or neurodegenerative (Huntington’s chorea, etc.) factors [3, 4]. Interest in immunological encephalopathies has increased in the last decade. Paraneoplastic limbic encephalitis (e.g., associated with anti-Hu antibodies), idiopathic autoimmune encephalitis (e.g., associated with anti-NMDAR- or anti-VGKC-LG1 antibodies), or Hashimoto encephalopathy (in the context of Hashimoto thyroiditis) can mimic schizophreniform and, in single cases, bipolar disorders, as well [3, 5–7]. These observations are highly relevant in understanding the etiology of psychiatric disorders because they show the necessity for a broad, organic diagnostic workup and for new treatment strategies with immunomodulatory medications, such as corticosteroids, intravenous immunoglobulins, or plasmapheresis [3, 8].

### Cerebrospinal fluid (CSF) characteristics in schizophreniform and bipolar disorders

Changes in the composition of cerebrospinal fluid (CSF) are detected frequently in patients with schizophreniform disorders [9–11]. We previously found mild pleocytosis in 3.4%, increased albumin quotients in 21.8%, elevated protein levels in 42.2%, and intrathecal immunoglobulin syntheses in 7.2% of a cohort with schizophreniform disorders [11]. Basic diagnostic CSF alterations were less frequent in bipolar patients; increased white blood cell counts were found in 1.6% of our bipolar cohort, increased albumin quotients in 12.8%, and intrathecal immunoglobulin synthesis in 4.8% [12]. Another study found increased lactate levels in 5 out of 15 patients with bipolar disorder [13]. In an earlier study, we detected a synthesis of polyspecific, intrathecal antibodies against different neurotropic infectious pathogens (*Toxoplasma gondii*, Herpes simplex virus types 1/2, cytomegalovirus, and Epstein–barr virus) in some patients with bipolar disorders [14].

### The MRZ reaction

The MRZ reaction (MRZR) is a marker for a polyspecific, antiviral, intrathecal, humoral immune response calculated as the antibody index (AI) directed against three neurotropic viruses of measles (M), rubella (R), and varicella zoster (Z) most commonly found in MS [15]. A positive MRZR was reported to be quite specific to multiple sclerosis (MS) [16, 17]. Elevated AIs (i.e., ≥ 1.5) usually occur due to intrathecal synthesis of antibodies against intrathecal pathogens. For example, in patients with Lyme neuroborreliosis, increased AIs demonstrate an intrathecal antibody synthesis against the pathogen *Borrelia burgdorferi* [18]. However, in case of a positive MRZR in MS patients, no causal virus DNA was detectable [19]; therefore, the MRZR has been interpreted as an autoimmune epiphenomenon of polyspecific B cell activation within the central nervous system [17, 20].

### Rationale for our study

Our recent findings of a potential polyspecific, antiviral, intrathecal, humoral synthesis against different neurotropic agents other than MRZ in bipolar patients, as well as frequent chronic inflammatory CSF alterations in schizophreniform patients, led to the question of whether a positive MRZR might be a marker of immunological encephalopathy in bipolar or schizophreniform disorders, similar to MS. We thus analyzed the MRZR in these patient cohorts and compared the findings with previously published data derived from two patient cohorts with MS and other inflammatory neurological disorders (OIND) [16, 21]. We hypothesized a positive MRZR in a subgroup of patients with potentially autoimmune-mediated schizophreniform and bipolar disorders.

## Participants and methods

This study was a part of a larger CSF project that received approval from the local ethics committee of the University of Freiburg (EK-Fr 609/14). CSF analysis was part of the routine clinical workup. As a screening procedure, lumbar punctures were routinely offered to patients with schizophreniform disorders. Bipolar patients were investigated unsystematically (i.e., in the case of signs of inflammation or neurodegeneration). Only those patients who provided written consent for lumbar puncture were included in the study.

### Study sample

We included 39 patients with schizophreniform syndromes and 39 patients with bipolar disorders. The schizophreniform cohort consisted of consecutively identified patients with schizophrenia, organic schizophrenia-like disorders, and schizoaffective [i.e. with (schizo)depressive or (schizo)manic episodes] disorders from which CSF was collected between 2016 and 2017. The bipolar cohort comprised bipolar or manic patients (i.e., with hypomanic, manic, depressive, mixed, or remitted episodes and organic bipolar disorders) in whom CSF was collected between 2006 and 2017. The patients were only one-time lumbar punctured in the course of the diagnostic routine examination, and single samples were analyzed retrospectively. Lumbar puncture was done using a sterile technique between the 3rd and 5th lumbar vertebrae (mostly between 4 and 5). The serum samples were taken on the same day. All samples were stored at − 80 °C after routine laboratory testing. The patient selection was unsystematic; only those patients with completed instrument-based diagnostics [i.e., electroencephalography (EEG), cerebral magnetic resonance imaging, laboratory, and CSF data] and those with sufficient residual serum/CSF material were included. Organic forms of schizophreniform or bipolar disorders were diagnosed after different serological tests, CSF, EEG and cerebral magnetic resonance imaging analyses. All patients with drug-induced schizophreniform and bipolar disorders were excluded. For comparison, we used a group of 48 patients with OIND (22 with neurosarcoidosis, 19 with autoimmune encephalitis, 7 with acute disseminated encephalomyelitis) and a group of 203 MS patients (100 with primary progressive MS, 103 with relapsing–remitting MS). The OIND and MS samples were reported in previous publications [21].

### MRZR measurement

The MRZR analyses were carried out at the Institute for Virology of the University of Freiburg. For the MRZR, the virus-specific AIs were determined for M, R, and Z. The M, R, and Z immunoglobulin (Ig) G (IgGspecific_(for M/R/Z)_) concentrations in the serum and CSF were measured with enzyme-linked immunosorbent assays (Serion classic, Würzburg, Germany) following the manufacturer’s instructions. The total Ig levels in the CSF and serum were analyzed nephelometrically (ProSpect System, Siemens, Munich, Germany) with the use of the same procedure established in previous studies [16, 21]. The virus-specific AIs for M, R, and Z were calculated with the quotient from CSF to serum antibody titers (QIgG[specific_(for M/R/Z)_]) and the reference to the relevant quotient of the total CSF/serum IgG (QIgG[total]) in relation to the age-corrected albumin quotient [22]. The calculation was performed using Reiber’s formula: AI = QIgG_[specific]_/QIgG_[total]_, if QIgG_[total]_ < Qlim, or AI = QIgG_[specific]_/Qlim, if QIgG_[total]_ > Qlim [23]. AIs ≥ 1.5 were defined as positive [16, 21, 23]. MRZR-1, which requires one positive AI, is less specific and of unclear significance. Therefore, a positive MRZR was defined by two or three increased AIs [16, 17, 21].

### Data handling and statistical analysis

All necessary information on our study cohort was transferred into the Statistical Package for the Social Sciences, version 22 database. Categorical variables (gender, MRZR) were compared with Pearson’s Chi squared test. The continuous variable of participants’ age was compared with two-sided independent sample t tests. A p < 0.05 was considered statistically significant.

## Results

### Demographic data of patients (Table 1)

The demographic parameters of our study cohort are summarized in Table 1. The patients with schizophreniform disorders differed significantly in age, but not in gender, from those in the OIND and MS groups. The bipolar patients differed in age and gender from those in the OIND group, but not from the MS cohort in either of these parameters.

**Table 1:** Demographic data of the study cohort

	Schizophreniform cohort (N = 39)	Bipolar cohort (N = 39)	Other inflammatory neurological disorders (N = 48^c^)	Multiple sclerosis (N = 203^c^)	Statistics
Mean age ± SD (Range in years)	32.3 ± 11.3 (18–75)	44.3 ± 14.7 (19–73)	51.8 ± 18.6 (4–84)	45.73 ± 12.128 (19–78)	p_1_ < 0.001 p_2_ < 0.001 p_3_ = 0.044 p_4_ = n.s.
Gender	19M: 20F	14M: 25F	29M: 19F	68M: 135F	p_1_ = n.s. p_2_ = n.s. p_3_ = 0.023 p_4_ = n.s.
Disorder catego-rization	Schizophrenia-like: 30^a^ Schizoaffective-like: 9	Hypomanic/manic episode^b^: 14 Depressive episode^b^: 20 Mixed episode: 4 Remitted: 1	Neurosarcoidosis: 22 Autoimmune encephalitis: 19 Acute disseminated encephalomyelitis: 7	Primary-progressive MS: 103 Relapsing–remitting MS: 100	

MS, multiple sclerosis; SD, standard deviation; M, male; f, female; p_1_, schizophreniform vs. OIND; p_2_, schizophreniform vs. multiple sclerosis; p_3_, bipolar vs. OIND cohort; p_4_, bipolar vs. MS cohort

^a^Six patients were diagnosed with organic schizophrenia-like disorders after diagnostic work-up

^b^Three patients turned out to have organic bipolar disorders after diagnostic work-up

^c^According to Hottenrott et al. [21]

### Frequency of MRZR in the study cohorts (Table 2)

Two patients with schizophreniform disorders showed a positive MRZR (5.1%; 2.6% in the whole psychiatric patient cohort). In four of the schizophreniform patients, one isolated AI (MRZR-1) was positive, which consisted of a specific reaction against varicella zoster. None of the bipolar controls showed a positive MRZR. Two bipolar patients had positive AIs for varicella zoster (MRZR-1). Four of the OIND patients had a positive MRZR, whereas 7 of these patients had one isolated positive AI (2 against rubella, 5 against varicella zoster). No relevant statistical differences were found between the frequency of positive MRZR in patients with schizophreniform and in those with bipolar disorders and OIND. The MS cohort showed a more frequently positive MRZR (99 of 203 patients: 48.8%) when compared to all other study groups (OIND, bipolar, and schizophreniform patients), and this difference was highly significant (p ≤ 0.001, Table 2). Focusing on only idiopathic psychiatric disorders (after the exclusion of organic schizophreniform or bipolar disorders) leads to increased overall prevalence rate in both patient groups of 2.9% (2 patients with positive MRZR out of 69 patients) and 6.1% of schizophreniform patients (2 patients with positive MRZR out of 33 patients). The exact findings of the MS subgroups were published in an earlier paper [21].

**Table 2:** MRZ reaction in the different patient groups

MRZR state	Schizophreniform cohort (N = 39)			Bipolar cohort (N = 39)			Other inflammatory neurological diseases (N = 48)			Multiple sclerosis (N = 203)		
	All AIs negative	MRZR-1	MRZR positive	All AIs negative	MRZR-1	MRZR positive	All AIs negative	MRZR-1	MRZR positive	All AIs negative	MRZR-1	MRZR positive
Number of patients (%)	33 (84.6)	4 (10.3)	2 (5.1)	37 (94.9)	2 (5.1)	0 (0)	37 (77.1)	7 (14.6)	4 (8.3)	48 (23.6)	56 (27.6)	99 (48.8)

MRZR-1 defined as having only one positive AI, MRZR defined as having 2 or 3 positive AIs

MRZR, MRZ reaction; AI, antibody index

### Characteristics of the MRZR-positive psychiatric patients (Table 3)

Two psychiatric patients showed a positive MRZR; both patients suffered from schizodepressive disorders. Neither of these two MRZR-positive patients displayed any neurological symptoms, and both had a normal CSF cell count without oligoclonal bands. Cerebral magnetic resonance imaging did not fulfill the MS criterion for dissemination in time and space, according to the 2010 revision of the McDonald diagnostic criteria [24]. The detailed characteristics of the MRZR-positive patients are presented in Table 3. Four schizophreniform patients had only one increased AI; these patients all suffered from isolated schizophrenia syndromes. One of these four patients (25%) was ultimately diagnosed with an organic schizophreniform disorder, but not due to MS. This male patient showed one isolated white matter (WM) lesion in the globus pallidus on the left side associated with an infectious mononucleosis in his youth. CSF analysis was normal. The two bipolar patients with one positive AI presented with a mixed and a manic episode during the time of lumbar puncture. None of these two patients was finally diagnosed with an organic bipolar disorder.

**Table 3:** Characteristics of MRZR-positive psychiatric patients

	Syndrome	Neurological examination	MRZR	CSF	cMRI	EEG	Other immunological testing
Patient 1: 34 year, male	Schizo-depressive syndrome	No neurological signs	M: 1.6 (↑) R: ↔ Z: 1.9 (↑)	Cell count: 3 (↔) Alb_Q_: 12 × 10^−3^ (↑)^a^ OCBs: negative	One unspecific white matter lesion right frontal	Normal	No antineuronal antibodies^b^, no rheumatological antibodies^c^, C3d slightly increased (9.6, reference < 9 mg/L). Increased anti-TG antibodies (150 IU/mL; reference < 115 IU/mL). Normal anti-TPO antibodies
Patient 2: 27 year, female	Schizo-depressive syndrome	No neurological signs	M: 1.7 (↑) R: ↔ Z: 1.5 (↑)	Cell count: 3 (↔) Alb_Q_: 3 (↔) OCBs: negative	Normal	Normal	No antineuronal antibodies^b^, no rheumatological antibodies^c^, C3d normal. Normal anti-TPO- and -TG-antibodies

MRZR, MRZ reaction; M, measles; R, rubella; Z, varicella zoster; CSF, cerebrospinal fluid; cMRI, cerebral magnetic resonance imaging; EEG, electroencephalography; Alb_Q_, albumin quotient; TG, thyroglobulin; TPO, thyroid peroxidase. ↔, normal; ↑, increased

^a^Age dependent reference: 6.5 × 10^−3^

^b^Including antibodies against intracellular onconeural antigens (*Yo*, *Hu*, *CV2*/*CRMP5*, *Ri*, *Ma1*/*2*, *SOX1*; measured in serum), against intracellular synaptic antigens (*GAD*, *amphiphysin*; in serum) and antibodies against neuronal cell surface antigens [*NMDAR*, *AMPA*-*R*, *GABA*-*B*-*R*, *VGKC*-*complex (LGI1*, *Caspr2)*; in CSF]

^c^Antinuclear, anti-neutrophil cytoplasmic, and antiphospholipid antibodies

## Discussion

In this pilot study, we investigated the MRZR in paired CSF and serum samples from 78 patients with bipolar and schizophreniform disorders. We were unable to verify our hypothesis of increased AIs suggestive of an autoimmune-driven intrathecal immune reaction in a relevant subgroup of psychiatric patients. Therefore, our results support the idea that the MRZR is rather specific to MS. Only two schizophreniform patients had a positive MRZR; both patients had schizodepressive disorders without clinical or paraclinical signs of MS [24].

### Earlier findings and clinical relevance of the MRZR

We analyzed the prevalence of a positive MRZR in a relevant psychiatric cohort. Earlier studies focused on patients with demyelinating disorders and OIND [17]. A recent review identified 30 studies that analyzed the MRZR. A positive MRZR was found in 458 of 724 (63.3%) patients with MS and in 19 of 754 control patients (2.5%). Therefore, the MRZR indicates a high cumulative specificity of 97.5% and a cumulative sensitivity of 63.3% for MS [17]. Our findings are in line with these observations, as we found a positive MRZR significantly more frequently in MS patients (48.8%) than in OIND (8.3%), schizophreniform (5.1%), or bipolar (0%) patients (p < 0.001). The prevalence rates in our schizophreniform cohort were comparable with those in the OIND group (5.1 vs. 8.3%; p = 0.557), indicating an association with immunological processes in a small schizophreniform subgroup. Both MRZR-positive schizophreniform patients had schizodepressive disorders without other signs of MS or brain inflammation. Schizodepressive disorders combine aspects of schizophrenia and depression and the distinction between schizophrenia and bipolar disorder is challenging [25]. Patients often show a relapsing–remitting course of the disorder comparable with MS. Male patient 1 had one single WM lesion and a relevant blood–brain barrier dysfunction. In combination with his increased titers of anti-thyroglobulin antibodies, this patient might suffer from Hashimoto encephalopathy [7]. However, no steroid treatment was given because not all clinical criteria were fulfilled (e.g., the patient had normal alpha-EEG and no subacute onset). The other female patient 2 had completely normal paraclinical diagnostics. Earlier research has shown that incipient cases of MS may present as psychosis [26]. Neither of the MRZR-positive patients in our cohort currently fulfill the McDonald diagnostic criteria [24]; however, only the future course can show whether these patients will develop symptoms of MS. In both patients, the AIs were only slightly increased between 1.5 and 1.9. Using a more stringent AI level > 2 as reference value would result in a normal MRZR in both patients, pointing to a potential unspecific mechanism. However, a larger study of 99 healthy volunteers found no positive MRZR in all these controls [27]. From our cohort, nine patients were ultimately diagnosed with organic schizophreniform (6 patients) and bipolar (3 patients) disorders. None of these patients had a positive MRZR. One of these patients had only one increased AI; this male patient showed one isolated WM lesion in the globus pallidus associated with a positive history of infectious mononucleosis in his adolescence. The other eight patients with organic psychiatric syndromes had normal AIs. Therefore, a single increased AI to MRZ antigens does not seem to be a strong indicator of an organic psychiatric pathophysiology. Taken together, our data do not support the idea that the MRZR could serve as a biomarker for a potentially autoimmune-driven inflammatory process affecting the CSF in patients with schizophreniform or bipolar disorders. Our findings correspond to the few MRZR-positive patients with OIND or other autoimmune disorders with central nervous involvement, implicating that the MRZR specificity for MS is high, but not 100%. However, the finding of a positive MRZR in a small subgroup of patients with schizophreniform disorders and our earlier reported CSF alterations are compatible with mild neuroinflammation, as proposed in the concept of mild encephalitis [28]. The investigation of further neurotropic agents (e.g., for Borna disease virus, Epstein–barr-virus) and other antineuronal antibodies would have led to the detection of a larger subgroup of patients with mild encephalitis [29].

### Limitations of this study

This study has several limitations. First, we did not assess a healthy control group for comparison. However, this would not have been ethically justified for this type of pilot study which requires CSF sampling. Therefore, we compared our findings with two previously published neurological control cohorts. Second, the study cohort was fairly small, and the gender ratio for bipolar disorder and the OIND group were not representative for the general population. However, our study was a retrospective pilot study of psychiatric patients who were not involved in CSF studies in most psychiatric hospitals. Therefore, in our opinion, the publication of these data from 78 patients, in conjunction with the two earlier published neurological patient cohorts with 251 patients, is still important. Third, the study cohort was inhomogeneous because primary, idiopathic, and secondary organic psychiatric patients were included. We used this approach because we hypothesized that precisely these subgroups of organic psychiatric syndromes would have a positive MRZR in case of a potentially immune-driven inflammatory process underlying the respective disorder. Fourth, the inclusion of patients was unsystematic, especially for the bipolar patients. However, in light of the negative results in this cohort, this procedure definitely did not lead to false-positive findings. Although the CSF from bipolar patients was investigated to exclude a potential organic cause, none of these patients had a positive MRZR. This implies that the MRZR is not a relevant biomarker for an autoimmune process in the CSF of patients with bipolar disorders. All in all, given the pilot nature of the scientific question explored in this clinical research, we believe that assessing such a question in retrospective analyses is justifiable, if not unavoidable. The alternative would have been to organize a large prospective study, which seems unjustifiable considering the resource input and potential output of such efforts. Nevertheless, we investigated a relevant research question following a logical path of reasoning, and our results are important in terms of planning endeavors for further research in the field.

## Conclusion

In summary, our study supports the high specificity of the MRZR for MS. A positive MRZR was much more frequent in MS than in bipolar and schizophreniform patients, and the difference was highly statistically significant. We detected only two patients with positive MRZR, and both patients suffered from schizodepressive episodes without further evidence of MS. The pathophysiological meaning of this finding remains elusive for the time being but would be compatible with mild encephalitis in these patients [28].

## Abbreviations

AI: antibody index
CSF: cerebrospinal fluid
EEG: electroencephalography
Ig: immunoglobulin
M: measles
MRZR: MRZ reaction
MS: multiple sclerosis
OIND: other inflammatory neurological disorders
R: rubella
WM: white matter
Z: varicella zoster
